# Plasma Apolipoprotein Levels Are Associated with Cognitive Status and Decline in a Community Cohort of Older Individuals

**DOI:** 10.1371/journal.pone.0034078

**Published:** 2012-06-11

**Authors:** Fei Song, Anne Poljak, John Crawford, Nicole A. Kochan, Wei Wen, Barbara Cameron, Ora Lux, Henry Brodaty, Karen Mather, George A. Smythe, Perminder S. Sachdev

**Affiliations:** 1 Brain and Aging Research Program, University of New South Wales, Sydney, Australia; 2 School of Psychiatry, University of New South Wales, Sydney, Australia; 3 Bioanalytical Mass Spectrometry Facility, University of New South Wales, Sydney, Australia; 4 School of Medical Sciences, University of New South Wales, Sydney, Australia; 5 Dementia Collaborative Research Centre, University of New South Wales, Sydney, Australia; Nathan Kline Institute and New York University School of Medicine, United States of America

## Abstract

**Objectives:**

Apolipoproteins have recently been implicated in the etiology of Alzheimer’s disease (AD). In particular, Apolipoprotein J (ApoJ or clusterin) has been proposed as a biomarker of the disease at the pre-dementia stage. We examined a group of apolipoproteins, including ApoA1, ApoA2, ApoB, ApoC3, ApoE, ApoH and ApoJ, in the plasma of a longitudinal community based cohort.

**Methods:**

664 subjects (257 with Mild Cognitive Impairment [MCI] and 407 with normal cognition), mean age 78 years, from the Sydney Memory and Aging Study (MAS) were followed up over two years. Plasma apolipoprotein levels at baseline (Wave 1) were measured using a multiplex bead fluorescence immunoassay technique.

**Results:**

At Wave 1, MCI subjects had lower levels of ApoA1, ApoA2 and ApoH, and higher levels of ApoE and ApoJ, and a higher ApoB/ApoA1 ratio. Carriers of the apolipoprotein E ε4 allele had significantly lower levels of plasma ApoE, ApoC3 and ApoH and a significantly higher level of ApoB. Global cognitive scores were correlated positively with ApoH and negatively with ApoJ levels. ApoJ and ApoE levels were correlated negatively with grey matter volume and positively with cerebrospinal fluid (CSF) volume on MRI. Lower ApoA1, ApoA2 and ApoH levels, and higher ApoB/ApoA1 ratio, increased the risk of cognitive decline over two years in cognitively normal individuals. ApoA1 was the most significant predictor of decline. These associations remained after statistically controlling for lipid profile. Higher ApoJ levels predicted white matter atrophy over two years.

**Conclusions:**

Elderly individuals with MCI have abnormal apolipoprotein levels, which are related to cognitive function and volumetric MRI measures cross-sectionally and are predictive of cognitive impairment in cognitively normal subjects. ApoA1, ApoH and ApoJ are potential plasma biomarkers of cognitive decline in non-demented elderly individuals.

## Introduction

It is estimated that 35.6 million people worldwide currently suffer from dementia [1]. Alzheimer’s disease (AD) is the most common cause of dementia and AD pathogenesis may affect the brains of patients for years or even decades before clinical symptoms are fully expressed. Mild cognitive impairment (MCI) has been proposed to describe the early phase of cognitive decline that precedes dementia. MCI patients progress to AD, vascular and other kinds of dementia [2]. Annual rates of conversion from MCI to dementia are reported to range from 2.7% [3] to 10–15% [4], [5]. People with MCI are more likely to develop AD than cognitively normal individuals [6].

The etiology of sporadic AD is not well understood, and vascular risk factors appear to play an important role [7]. A history of vascular disease including heart disease and cerebrovascular disease has a negative impact on cognition in old age [8]. High serum total cholesterol at midlife is a risk factor for AD and other dementia types in later life [9], [10], and is also directly associated with higher risk of dementia mortality [11]. Clinical and epidemiological studies also support a strong relationship between AD and cardiovascular disease (CVD) risk factors such as high density lipoprotein (HDL) levels, low density lipoprotein (LDL) levels and the presence of atherosclerosis and hypertension [12]. Even though there is no consistent evidence for the role of cholesterol lowering agents in AD treatment, some studies suggest that they reduce the incidence of AD [13], [14].

**Figure 1:**
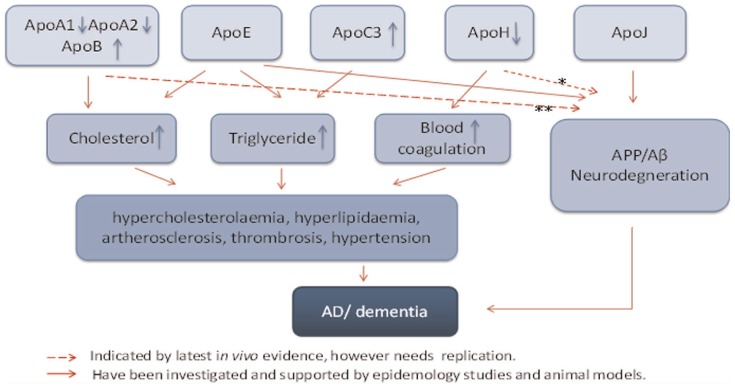
Potential pathophysiological mechanisms involving apolipoproteins in Alzheimer’s disease. Literature evidence: *, Katzav, Faust-Socher et al. 2011; George and Erkan 2009. **, Lewis, Cao et al. 2010; Roher, Maarouf et al. 2009; Martins, Berger et al. 2009; Bereczki, Bernat et al. 2008.

Apolipoproteins (Apo) are a group of proteins related to cholesterol and lipid metabolism [15], [16], and recent findings indicate that apolipoproteins might also be involved in neurodegenerative processes [17], [18], [19], [20] (figure 1). A transgenic mouse model overexpressing amyloid precursor protein (APP) and presenilin 1 (PS1) was found to have increased plasma ApoJ (clusterin) levels, as well as amyloid and ApoJ co-localization in plaques [20]. ApoA1 and ApoA2 are major components of HDL, and are involved in transport of cholesterol to the liver [15]. A triple transgenic mouse model (overexpressing mutant forms of APP, PS1 and ApoA1) showed that overexpression of ApoA1 prevented the development of age-related learning and memory deficits despite continued Aβ deposition [18]. Furthermore, Kawano *et al* found lower plasma levels of ApoA1 and ApoA2 in Japanese patients with late-onset non-familial AD [21]. Bereczki *et al* showed that overexpression of human ApoB in the serum of transgenic mice caused the formation of amyloid plaques and extensive neuronal death [22] and two studies have found significantly higher levels of ApoB in the serum of AD subjects [23], [24]. Furthermore, Scacchi *et al* [25] have demonstrated a link between the *APOB* EcoR1 R+R genotype and higher total cholesterol and LDL cholesterol relative to R+R+ homozygotes. However, apart from this handful of disparate studies, little is known about the role of ApoB at preclinical stages of dementia such as MCI. ApoC3 is a component of very low density lipoprotein (VLDL) and delays the breakdown of triglycerides, leading to development of hypertriglyceridemia and increased risk of atherosclerosis [26].

The *APOE* ε4 allele is a significant genetic risk factor for sporadic AD [27], [28]. In the periphery, ApoE aids transport of triglycerides, phospholipid, cholesterol esters and cholesterol into cells [16]. However, the literature is conflicted on the direction of change in levels of plasma ApoE protein in AD as well as the effect of the *APOE* polymorphism on protein levels. A few studies have reported significantly lower ApoE protein in patients with AD [29], [30], [31], [32], [33], while several other studies show increased levels [34], [35], [36]. Similarly the literature on the relationship between *APOE* ε4 allele and plasma ApoE levels is limited and controversial with some studies showing increased apoE protein levels in *APOE* ε4 allele carriers [31], [33], [35], while other studies observe the opposite [29], [30]. Furthermore, there is evidence in the literature that the *APOE* polymorphism may affect the expression level of other apolipoproteins, such as ApoB, as well [16], however fleetingly little work has been done on this topic. Consequently additional studies are needed to help resolve the question of what role, if any, genetic variance has on plasma apolipoprotein levels.

ApoH is involved in diverse physiological processes, including lipid metabolism [37], however, little is known about the role if any that this protein may play in MCI or dementia. Only a single study is available, showing elevated ApoH in the CSF of several dementia subtypes [38]. In recent studies, allele variants of *APOJ* (*CLU*,clusterin) were shown to be associated with increased risk of AD [39], [40]. Higher plasma ApoJ levels are reported to be associated with atrophy of the entorhinal cortex and faster cognitive decline in AD [20], as well as involved in clearance of amyloid beta (Aβ) [41].

Therefore, these seven apolipoproteins, ApoA1, ApoA2, ApoB, ApoC3, ApoE, ApoH and ApoJ, were chosen for the analysis of plasma in MCI and cognitively normal subjects in a community based study. The aims of the present study were, to determine if plasma apolipoproteins are abnormal in MCI subjects at an early stage of cognitive decline, and to establish if baseline apolipoprotein levels are predictive of cognitive impairment in cognitively normal subjects over a two year period. Finally, we wished to examine the effect of *APOE* ε4 allele on plasma ApoE levels, correlations of apolipoproteins with age and with lipid profiles, including total cholesterol, HDL-cholesterol, LDL-cholesterol and triglycerides, and the association of lipid profile with cognitive decline. We tested the hypothesis that apolipoproteins involved in the transportation of cholesterol and triglycerides are risk factors for cognitive impairment, and cognitive decline in elderly non–demented individuals.

## Methods

### Ethics Statement

The procedures of this study were approved by Human Research Ethics Committee of the University of New South Wales on human experimentation, and written informed consent was obtained from all participants involved in this study.

### Subjects and Samples

Plasma samples were from Wave 1 (baseline) of the Sydney Memory and Ageing study (MAS), described in detail in a previously published paper [42], which comprised a cohort of non-demented elderly individuals drawn randomly from the community through the electoral roll. Individuals aged 70–90 years (n = 8914) were initially invited to participate MAS and 7142 either did not respond to the letter or declined to participate [42]. Of the remaining 1772 contacted by telephone to confirm eligibility, 735 were either ineligible or declined after further information about the study [42]. The final sample comprised 1037 individuals who received detailed Wave 1 assessment documentation [42]. Of these, 943 provided a blood sample, and magnetic resonance imaging (MRI) brain scans were performed on 544 individuals.

Participants were excluded if they had a previous diagnosis of dementia, psychotic symptoms or a diagnosis of schizophrenia or bipolar disorder, multiple sclerosis, motor neuron disease, developmental disability, progressive malignancy (active cancer or receiving treatment for cancer, other than prostate non-metastasized, and skin cancer), or if they had medical or psychological conditions that may have prevented them from completing assessments. Participants were excluded if they had a Mini-Mental State Examination (MMSE) [43] score of *<*24 adjusted for age, education and non-English speaking background [44] at study entry, or if they received a diagnosis of dementia after comprehensive assessment.

Participants received a detailed neuropsychological evaluation, and a subset had MRI brain scans at Waves 1 and 2, two years apart. A subgroup of 664 subjects was included in this study, of which the Wave 1 characteristics are shown in table 1. Blood samples were obtained via venipuncture after overnight fasting, and transferred into ethylenediaminetetraacetic acid (EDTA) coated tubes. Blood samples were centrifuged (1500 g) at room temperature for 15 minutes. Supernatant plasma was collected and aliquoted into polypropylene tubes. Plasma samples were stored at -80 °C, and thawed immediately before assay.

**Table 1:** Participant Wave 1 demographic information and apolipoprotein levels.

	Normal n = 407	MCI n = 257	p	P(FDR corrected)
**Age, years**	77.9±4.5	78.8±4.7	**0.01**	–––
**Male, n, %**	175(43%)	126 (49%)	0.13	–––
**Years of education**	11.6±3.5	11.3±3.4	0.30	–––
***APOE*** ** ε4 carrier, n, %**	84 (20.6%)	72 (28.0%)	**0.03**	–––
**Hypolipidaemic medications, n,%**	205 (49.9%)	124(47.5%)	**0.55**	
**ApoA1 (mg/ml)**	2.97±1.28	2.70±1.12	**0.01**	**0.013**
**ApoA2 (µg/ml)**	158.63±45.78	148.50±47.63	**0.003**	**0.008**
**ApoB (mg/ml)**	1.86±0.53	1.98±0.68	0.07	0.08
**ApoC3 (µg/ml)**	64.04±23.59	60.12±24.13	0.08	0.08
**ApoE (µg/ml)**	36.30±20.40	39.86±22.00	**0.01**	**0.013**
**ApoH (µg/ml)**	171.40±43.07	156.44±45.10	**0.00002**	**0.00008**
**ApoJ (µg/ml)**	108.97±27.43	119.98±33.03	**0.000006**	**0.00008**
**ApoB/ApoA1**	0.73±0.38	0.86±0.47	**0.005**	**0.01**

Data are presented as mean±SD.

Covariates in ANCOVA for effects of apolipoproteins: age, sex, years of education, *APOE* **ε**4 carrier status, hypolipidaemic medications.

### Clinical Evaluation

Table S1 in the supplementary data lists cognitive domains that each neuropsychological test was allocated to for the purpose of calculating domain scores. These domains were attention/processing speed, memory, verbal memory, language, visuo-spatial ability and executive function, as detailed in previously published article [42]. Domain scores were formed as the average of the Z-scores of their component tests.

The diagnosis of MCI was based on international consensus criteria [45], as follows: (a) complaint of decline in memory or other cognitive function which may be self- or informant-reported; (b) cognitive impairment on objective testing, i.e. not normal for age as determined by performance on at least one test measure 1.5 SDs or more below published normative values (or comparable standardized score compared to age and/or education-matched samples); (c) participants did not have a pre-existing diagnosis of dementia on entry to the study, had an adjusted MMSE score of ≥24 and did not meet DSM-IV criteria [46] for possible or probable dementia; (d) essentially normal function or minimal impairment in instrumental activities of daily living (IADLs) defined by a total average score *<*3.0 on the informant rated Bayer Activities of Daily Living Scale (B-ADL) [47].

Clinical Dementia Rating (CDR) [48] scores were obtained on all participants for both Waves. CDR of 0 indicated normal whereas CDR 0.5 and CDR 1 indicated cognitive impairment (MCI or very mild dementia) and mild AD dementia, respectively [4].

### APOE Genotyping

Genomic DNA was extracted from peripheral blood leukocytes or saliva samples using standard methods. Genotyping of two apolipoprotein E gene (*APOE*) single nucleotide polymorphisms (SNPs) was undertaken to determine the *APOE* genotype, which is comprised of three alleles (ε2, ε3, ε4). Two separate Taqman genotyping assays (Applied Biosystems Inc. [ABI], Foster City, CA) were used to genotype the two *APOE* SNPs, NCBI *rs7412* and NCBI *rs429358.* The SNP NCBI *rs7412* distinguishes the ε2 allele from the ε3/ε4 alleles whilst the SNP NCBI *rs429358* differentiates the ε4 allele from the ε2/ε3 alleles [49]. *APOE* ε4 carrier indicates carrying at least one *APOE* ε4 allele.

### Quantification of Apolipoproteins in Wave 1 Plasma Samples

Plasma apolipoprotein concentrations were assayed using multiplex fluorescent immunoassay kits (WideScreen™ Human CVD Panel 1; Novagen, EMD Chemicals Inc, WI). The xMAP platform used here was based on the Rules Based Medicine (RBM) fluorescent beads and antibody pairs. These are sensitive, specific and widely used reagents, sourced by numerous manufacturers and data collected using xMAP multiplex beads are widely reported in the literature in studies where simultaneous assay of multiple plasma proteins, including apolipoproteins, are performed. Some recent examples of published work which has made use of human xMAP multiplex technology include; [38], [50], [51]. Plasma samples (5 µL) were diluted 1∶2500 in dilution buffer, and manufacturer’s instructions were followed, with the additional precaution of handling the fluorescent beads in a darkened room. Samples were run in duplicate using a Bioplex system (Luminex 100, BioRad, Hercules, CA) Bio-Plex Manager 4.0 using a 5 parameter logistic regression model. The average intra- and inter-assay CVs were <7.0% and <17.9%, respectively. The range of standard concentrations is provided in (Table S2).

### Quantification of Lipid Profile in Wave 1 Plasma Samples

Total cholesterol, HDL-cholesterol and triglycerides were measured in heparin plasma aliquots using a Beckman LX20 Analyzer using a timed-endpoint method (Fullerton, CA). This direct HDL-cholesterol method requires no off-line pre-treatment steps. LDL-cholesterol was estimated using the Friedewald equation (LDL-cholesterol = total cholesterol - HDL-cholesterol - triglycerides/2.2). These assays were conducted in an independent laboratory of South Eastern Area Laboratory Services.

### Acquisition of Neuroimaging Data

All participants were invited to undergo an MRI scan, and those who agreed were screened for contra-indications (pacemaker, metallic implant or foreign bodies, cochlear implants, ferromagnetic homeostatic clips, claustrophobia). A subgroup (n = 376) was included in this study at Wave 1, and 282 subjects at Wave 2. Subjects were scanned using a Philips 3 T Intera Quasar scanner (Philips Medical Systems, Best, Netherlands). The main parameters for T1-weighted 3D structural MRI were: TR = 6.39 ms, TE = 2.9 ms, flip angle = 8°, matrix size = 256×256, FOV = 256×256×190, and slice thickness = 1 mm with no gap between; yielding 1×1×1 mm3 isotropic voxels. The T2-weighted fluid attenuated inversion recovery (FLAIR) sequence was acquired with TR = 10000 ms, TE = 110 ms, TI = 2800; matrix size = 512×512; slice thickness = 3.5 mm with no gap between slices, yielding spatial resolution of 0.488×0.488×3.5 mm3/voxel.

### Statistical Analysis

All statistical analyses were performed using SPSS Version 18.0 (SPSS Inc., Chicago, IL). Differences between normal and MCI groups on categorical variables (sex and *APOE* **ε**4 status) were evaluated using Chi squared tests, and t-tests were used for examining differences between these groups on age and years of education. Group differences between apolipoprotein levels were assessed using analysis of covariance (ANCOVA), with age, sex, years of education and *APOE* **ε**4 status entered as covariates. For these analyses, the distributions of apolipoprotein levels were inspected and, when necessary, transformed to more closely approximate the normal distribution. As a result, ApoA1, ApoA2, ApoE, ApoJ and ApoB/ApoA1 ratio were log transformed and ApoB, ApoC3 and ApoH levels were square-root transformed. Partial correlations were used to examine the relationships of the apolipoproteins with lipid levels, cognitive domain composite scores and brain volumetric measures. Control variables were age and sex for correlations with lipids; age, sex, years of education and *APOE* **ε**4 status for correlations with neuropsychological measures; and age, sex and intracranial volume (ICV) for correlations with brain volumes. An alternative approach was to apply a non-parametric test to the raw (non-transformed) data. Consequently we also checked the data using the Spearman Rank Correlation, however the main results did not change.

To assess if plasma apolipoproteins levels predicted cognitive decline in cognitively normal participants, logistic regression analysis was performed, using only those participants with a CDR = 0 at Wave 1, and a CDR of >0 (versus CDR = 0) at Wave 2 as the dependent variable. We used the z-scores of apolipoproteins as independent variables, so that the odds ratios (ORs) reflect the ratio of odds when values of apolipoproteins increase by one standard deviation (SD). Age, sex, years of education and *APOE* **ε**4 status were included in this analysis as control variables. Also included as a control variable was a CVD risk index which we calculated based on the regression model of the Framingham Heart Study [52]. This index used the following MAS Wave 1 variables: current smoking status, diabetic status, systolic blood pressure, total cholesterol level, HDL level and current use of antihypertensive medication. When blood analyses were unavailable (n = 3), Body Mass Index was used instead of cholesterol and HDL data.

Linear regression models were used to examine whether Wave 1 apolipoprotein levels were associated with brain volumetric changes from Wave 1 to Wave 2, including changes in CSF volume, grey matter, white matter and hippocampal volume, and brain atrophy between the two waves. The brain atrophy measure during follow-up was calculated as the changes of brain volume (sum of grey matter and white matter volumes) divided by ICV. Transformed apolipoprotein levels were used as independent variables and age and sex were covariates. ICV was also used as a control variable for brain volume variables, except for the atrophy measure.

P-values, corrected for multiple testing using the false discovery rate (FDR) method, were used when evaluating the statistical significance of results [53]. P values in the ANCOVA, partial correlation and logistic regression were corrected by FDR. After FDR correction, P<0.05 was considered as significant level.

## Results

### Demographic Characteristics and Apolipoprotein Levels at Wave 1

The Wave 1 demographic information on cognitively normal and MCI participants is shown in table 1. MCI subjects were slightly older and more likely to be *APOE* **ε**4 carriers. No statistical differences were noted in gender and years of education between MCI and cognitively normal subjects. ApoE and ApoJ had weak but significantly positive correlations with age at Wave 1 (ApoE: r = 0.11, p = 0.003; ApoJ: r = 0.09, p = 0.02). We studied the association of all seven apolipoproteins assayed in this study with *APOE ε4* genotype (table 2). After adjusting for age and gender, *APOE ε*4 homozygous carriers have significantly lower plasma ApoE level than non *APOE ε*4 carriers or *APOE ε*4 heterozygote carriers. ApoE levels are also positively correlated with age. Furthermore, plasma levels of ApoB are significantly increased and ApoC3 significantly decreased in *APOE ε*4 heterozygote carriers. Plasma levels of ApoH are close to significantly decreased (p = 0.08) in *APOE ε*4 heterozygote carriers, and are in fact significantly decreased relative to non-carriers when the data for heterozygotes and homozygotes are pooled (table 2).

**Table 2:** Apolipoprotein levels in different *APOE ε*4 carrier groups.

	Non *APOE ε*4 carrier	*APOE ε*4 heterozygote carrier	*APOE ε*4 homozygous carriers	F	p
N	508	144	12		
**ApoA1 mg/ml**	2.86±1.22	2.91±1.26	2.64±1.21	0.62	0.54
**ApoA2 µg/ml**	155.13±47.29	151.95±43.64	170.27±58.37	0.70	0.50
**ApoB mg/ml**	1.86±0.59	2.04±0.62*	2.10±0.31	5.11	0.006
**ApoC3 µg/ml**	64.04±24.44	57.80±21.11	54.88±22.76	4.31	0.01
**ApoE µg/ml**	41.16±21.44	26.94±14.83**	19.06±17.54**§	56.44	<0.0005
**ApoH µg/ml**	167.66±44.78	159.95±42.87	147.00±41.50	2.56	0.08#
**ApoJ µg/ml**	113.64±30.62	111.76±28.33	113.66±34.88	0.07	0.94

Statistics details: ANCOVA, Post-hoc: Bon Ferroni Covariates: age, sex.

* Compared to non*APOE* ε4 carrier, p<0.05, **Compared to non*APOE* ε4 carrier, p<0.0005.

§ Compared to *APOE* ε4 heterozygote carrier, p<0.0005.

# When heterozygous and homozygous carriers are pooled and compared with non *APOE* ε4 carriers the ApoH values are statistically significant (n = 156, mean = 158.95±42.78, F = 4.23, p = 0.04). Pooling of data from heterozygous and homozygous carriers makes no difference to the statistical outcomes for any of the other apolipoproteins, though the significant p values all become even slightly lower.

After adjusting the p values using the FDR method, MCI subjects had lower levels of ApoA1 (F = 6.34, p = 0.013), ApoA2 (F = 9.20, p = 0.008) and ApoH (F = 18.78, p = 0.00008), and higher levels of ApoE (F = 6.06, p = 0.013), ApoJ (F = 20.76, p = 0.00008) and ApoB/ApoA1 (F = 8.10, p = 0.01).

### Partial Correlations Amongst Apolipoproteins and with Lipid Levels

Most correlations between apolipoproteins were weak to moderate (table 2; 0.10<r<0.50, p<0.001). Several correlations (those between ApoA1 and ApoA2, ApoA2 and ApoC3, ApoA2 and ApoH, ApoA2 and ApoB/ApoA1) were relatively strong with values greater than 0.50 (table 3). Correlations between apolipoproteins, and lipid levels varied from near zero to a moderately high value of 0.60 between ApoA1 and HDL-cholesterol, and 0.53 between ApoE and triglycerides (p<0.001) (table 3). No significant correlations were shown between ApoJ and total cholesterol, HDL-cholesterol, LDL-cholesterol or triglycerides (p>0.05) (table 3).

**Table 3:** Partial correlations between apolipoproteins and lipid profiles.

	ApoA1	ApoA2	ApoB	ApoC3	ApoE	ApoH	ApoJ	ApoB/ApoA1
**ApoA1**	–	0.60***	0.01	0.39***	−0.05	0.35***	0.16***	–
**ApoA2**	0.60***	–	–0.04	0.57***	0.09	0.50***	0.23***	–0.50***
**ApoB**	0.01	−0.04	–	–0.08	0.05	−0.10	0.23***	–
**ApoC3**	0.39***	0.57***	–0.08	–	0.43***	0.47***	0.32***	–0.36***
**ApoE**	−0.05	0.09	0.05	0.43***	–	0.25***	0.36***	0.05
**ApoH**	0.35***	0.50***	−0.10	0.47***	0.25***	–	0.30***	−0.34***
**ApoJ**	0.16***	0.23***	0.23***	0.32***	0.36***	0.30***	–	−0.01
**ApoB/ApoA1**	–	−0.50***	–	−0.36***	0.05	−0.34***	−0.01	–
**HDL-cholesterol**	0.60***	0.22***	−0.001	0.13**	−0.24***	0.03	0.01	−0.48***
**LDL-cholesterol**	0.01	0.07	0.29***	0.08	0.06	0.06	0.03	0.16***
**Total cholesterol**	0.24***	0.18***	0.25***	0.28***	0.10*	0.11**	0.05	−0.04
**Triglycerides**	−0.22***	0.06	−0.02	0.49***	0.53***	0.19***	0.08	0.16***

* p<0.05, **p<0.01, *** p<0.001(FDR corrected p values) Covariates: age, sex.

### Plasma Apolipoproteins and Neuropsychological Performance at Wave 1

Table 4 shows the partial correlation of plasma apolipoproteins with global cognitive and cognitive domain scores at Wave 1. ApoH levels were positively correlated with global cognition (r = 0.15, p = 0.001), attention/processing speed (r = 0.11, p = 0.04) and executive function (r = 0.15, p = 0.006). ApoJ levels were negatively correlated with scores of global cognition (r = −0.13, p = 0.04) and attention/processing speed (r = −0.11, p = 0.04). ApoE levels were negatively correlated with total memory (r = −0.11, p = 0.04) and verbal memory domain scores (r = −0.11, p = 0.04). ApoA2 levels were positively correlated only with attention/processing speed (r = 0.12, p = 0.04). Though the correlations at Wave 1 were modest, they remained significant after adjusting for FDR. Scatter plots of significant correlations are presented in (figure S1, S2, S3, S4, S5, S6, S7).

**Table 4:** Partial correlations between apolipoprotein levels and global cognition/cognitive domain composite scores at Wave 1.

		ApoA1	ApoA2	ApoB	ApoC3	ApoE	ApoH	ApoJ	ApoB/ApoA1
**Global cognition**	**r**	0.04	0.07	−0.06	0.02	−0.07	0.15**	−0.13*	−0.05
**Attention/processing speed**	**r**	0.07	0.12*	−0.05	0.04	−0.05	0.11*	−0.11*	−0.05
**Memory**	**r**	0.01	0.03	−0.03	−0.04	−0.11*	0.09	−0.09	−0.03
**Verbal memory**	**r**	0.01	0.01	−0.02	−0.05	−0.11*	0.08	−0.07	−0.03
**Language**	**r**	−0.01	0.02	−0.07	0.003	−0.009	0.09	−0.06	−0.03
**Visuo-spatial**	**r**	0.01	0.03	−0.01	−0.007	−0.05	0.07	−0.07	−0.01
**Executive function**	**r**	0.06	0.05	−0.03	0.02	−0.07	0.15**	−0.04	−0.04

Subject numbers, n = 657.

* p<0.05; **p<0.01 (FDR corrected p values, see text).

Covariates: age, sex, years of education, *APOE* **ε**4 carrier status.

### Plasma Apolipoproteins and Brain Volumes at Wave 1

Wave 1 apolipoprotein levels were correlated with grey matter and CSF volume (table 5). ApoC3, ApoE and ApoJ were negatively correlated with grey matter volume (ApoC3: r = −0.14, p = 0.038; ApoE: r = −0.14, p = 0.038; ApoJ: r = −0.15, p = 0.036) and positively correlated with CSF volume (ApoE: r = 0.16, p = 0.036; ApoJ: r = 0.14, p = 0.038). The ApoB/ApoA1 ratio was also negatively correlated with hippocampal volume (r = −0.13, p = 0.04). These correlations are modest but statistically significant. Scatter plots of significant correlations are presented in (figure S8, S9, S10, S11, S12, S13).

**Table 5:** Partial correlation between apolipoprotein levels and brain volumes at Wave 1.

		ApoA1	ApoA2	ApoB	ApoC3	ApoE	ApoH	ApoJ	ApoB/ApoA1
**Grey matter**	**r**	−0.01	−0.08	−0.03	–0.14*	−0.14*	−0.06	−0.10*	−0.07
**White matter**	**r**	0.08	0.03	0.003	–0.02	−0.05	0.01	−0.01	−0.05
**CSF volume**	**r**	−0.03	0.05	0.02	0.11	0.16*	0.05	0.14*	0.08
**Hippocampus**	**r**	0.08	0.05	−0.06	–0.03	–0.02	0.01	−0.03	−0.13*

Subject numbers, n = 377.

* p<0.05 (FDR corrected p values, see text).

Covariates: age, sex, intracranial volume.

### Apolipoprotein Levels to Predict Future Cognitive Decline

We investigated if apolipoprotein levels at Wave 1 could predict conversion from cognitively normal (CDR = 0) at Wave 1 to cognitively impaired (CDR >0) at Wave 2. For this analysis, 517 participants had a CDR = 0 at Wave 1 and of these 149 participants had a CDR greater than 0 at Wave 2. Individuals who went from CDR 0 to CDR >0 were classified as *converters*, and those remaining CDR 0 at Wave 2 were considered *nonconverters*. Group characteristics are described in table 6. The converting rate to CDR cognitively impaired is 14.41% annually.

**Table 6:** Demographic characteristics of nonconverters and converters at Wave 1.

	Nonconverters (Wave 2 CDR = 0)	Converters (Wave 2 CDR >0)	p
N	368	149	
**Age, mean(SD),y**	77.3±4.1	79.2±4.7	**<0.0001**
**Male, n,%**	140, 38%	85, 43%	**<0.0001**
**Years of education**	11.8±3.4	10.7±3.4	**0.001**
***APOE*** ** ε4 carrier, n, %**	65, 17.9%	42, 28.6%	**0.007**
**MMSE score, mean(SD)**	28.5±1.2	27.8±1.4	**<0.0001**

Table 7 shows the results of a series of logistic regression analyses predicting cognitive decline with conversion from CDR = 0 to CDR >0, with each apolipoprotein entered singly into each model together with control variables age, sex, years of education and *APOE* **ε**4 carrier status. Results are also shown for a second series of analyses that also included the CVD risk index as an additional control variable. This revealed that ApoA1, ApoA2, ApoH and ApoB/ApoA1 ratio significantly predicted conversion, both with and without the CVD risk index being included in the models. A decrease of one standard deviation of ApoA1, ApoA2, ApoH and an increase of one standard deviation of ApoB/ApoA1 increased the risk of conversion to CDR >0 (ApoA1: OR = 0.65, P = 0.016; ApoA2: OR = 0.76, P = 0.04; ApoH: OR = 0.77, P = 0.04; ApoB/ApoA1: OR = 1.46, P = 0.04). In summary, participants with lower ApoA1, ApoA2, ApoH levels or higher ApoB/ApoA1, at Wave 1 had a higher risk of future cognitive decline.

**Table 7:** Logistic regression analyses for the prediction of conversion from CDR 0 at Wave 1 to CDR >0 at Wave 2 from Wave 1 plasma apolipoprotein levels.

	Models without CVD risk index includedas a control variable					Models with CVD risk index included as a control variable					Models with CH, TG, HDL,LDL includedas control varaible				
	OR	95%CI	Wald	P	p (FDR )	OR	95%CI	Wald	p	P (FDR)	OR	95%CI	Wald	p	P (FDR)
**ApoA1**	0.61	0.46–0.80	12.82	**0.0003**	**0.0024**	0.65	0.49–0.86	9.18	**0.002**	**0.016**	0.56	0.41–0.78	11.98	**0.001**	**0.008**
**ApoA2**	0.73	0.58–0.91	7.61	**0.006**	**0.016**	0.76	0.61–0.96	5.27	**0.022**	**0.04**	0.72	0.57–0.91	7.45	**0.006**	**0.016**
**ApoB**	1.17	0.93–1.47	1.74	0.19	0.27	1.15	0.91–1.45	1.33	0.25	0.33	1.15	0.91–1.47	1.36	0.24	0.38
**ApoC3**	0.87	0.69–1.08	1.64	0.20	0.27	0.87	0.69–1.09	1.51	0.22	0.33	0.76	0.57–1.00	3.76	0.52	0.66
**ApoE**	1.16	0.89–1.50	1.23	0.27	0.31	1.13	0.86–1.49	0.77	0.38	0.43	1.06	0.76–1.46	0.10	0.75	0.75
**ApoH**	0.76	0.61–0.94	6.26	**0.012**	**0.024**	0.77	0.62–0.96	5.50	**0.019**	**0.04**	0.74	0.59–0.93	6.90	**0.009**	**0.018**
**ApoJ**	1.06	0.85–1.32	0.29	0.59	0.59	1.08	0.86–1.35	0.44	0.51	0.51	1.07	0.85–1.33	0.31	0.58	0.66
**ApoB/ApoA1**	1.60	1.–2.11	10.92	**0.001**	**0.004**	1.46	1.09–1.95	6.53	**0.011**	**0.04**	1.61	1.18–2.21	8.90	**0.003**	**0.012**

Covariates: age, sex, years of education and *APOE* **ε**4 carrier status. CVD risk index was also included as a covariate in the series of analyses shown on the left side of table.

¶ CH, cholesterol; TG, triglycerides; HDL, high density lipoprotein; LDL, low density lipoprotein.

To study which apolipoproteins had the highest independent predictive value, the z-scores of all apolipoprotein measures were entered together into a stepwise logistic regression (probability of F for entry = 0.05, and for removal = 0.10), while adjusting for age, sex, years of education, *APOE* **ε**4 carrier status and CVD risk index. Only ApoA1 showed a statistically significant effect on cognitive decline from CDR = 0 to CDR >0 after controlling for the effects of the other apolipoproteins, and also age, sex, years of education, *APOE* **ε**4 carrier status and CVD risk index (OR = 0.63, 95%CI = 0.48–0.84, P = 0.001).

### Relationship of Apolipoproteins with Brain Volume Changes During Follow-up

With regard to the relationship between apolipoproteins and brain neuroimaging changes, a slightly different pattern emerged. For this analysis, 232 participants had neuroimaging data for both waves. Linear regression models showed that increased ApoC3 levels were associated with a decrease in grey matter volume (β = 0.19, p = 0.04), and increased ApoJ levels were associated with a decrease in white matter volume (β = 0.16, p = 0.03) after two years follow-up. Lower ApoA1 levels showed a weak but not significant association with an increase in brain atrophy (β = −0.15, p = 0.09). There were no associations between apolipoprotein levels and increase in CSF volume or decrease in hippocampus volume.

### Lipid Profile and Cognitive Decline

The lipid profiles at Wave 1 were analysed to investigate if they could predict conversion from cognitively normal (CDR = 0) at Wave 1 to cognitively impaired (CDR>0) at Wave 2. However, none of the measures examined was associated with cognitive impairment during follow-up; total cholesterol (OR = 1.06, p = 0.94), HDL-cholesterol (OR = 0.87, p = 0.71), LDL-cholesterol (OR = 0.95, p = 0.95) and triglycerides (OR = 1.02, p = 0.95). Furthermore, including lipid profile as covariates did not affect the ability of ApoA1, ApoA2 and ApoH levels and ApoB/ApoA1 ratio to predict cognitive impairment (table 6).

## Discussion

In this study, we report for the first time detailed measurements of multiple apolipoproteins in the plasma of elderly non-demented subjects, and relate them to cognition. Our salient findings are that MCI subjects at Wave 1 had lower levels of ApoA1, ApoA2, ApoH and higher levels of ApoE, ApoJ and the ApoB/ApoA1 ratio. Furthermore ApoE, ApoC3 and ApoH show significant downward trends in *APOE* ε4 allele carriers, whereas ApoB levels are significantly increased in ε4 carriers. Two years later, lower ApoA1, ApoA2, ApoH levels and higher ApoB/ApoA1 ratio were associated with cognitive decline in cognitively normal participants after adjusting for age, sex, years of education, *APOE* **ε**4 carrier status and CVD risk index (See the summary in table 8). Furthermore, lipid profile was not related with cognitive decline, and did not affect the association of apolipoproteins with cognitive impairment.

**Table 8:** Summary of correlations between apolipoproteins and cognitive impairment, cognitive decline and MRI changes over 2 years.

	Cognitiveimpairment	Cognitive decline over2 years	MRI lower volumeat Wave 1	MRI volume decreaseover 2 years
**ApoA1 - low**	+	+		
**ApoA2 - low**	+	+		
**ApoB - high**				
**ApoC3 - high**			+	+
**ApoE – high**	+		+	
**ApoH – low**	+	+		
**ApoJ - high**	+		+	+
**ApoB/ApoA1 high**	+	+	+	

+ Significantly indicates relationship in direction of pathology.

Our results therefore indicate that apolipoproteins are associated with cognitive decline independently of the effect of lipid profile. The pathomechanisms underlying this relationship still remain unclear. A few clinical studies have revealed that some apolipoproteins are deregulated in AD patients [20], [54], and work on transgenic animal models suggests that some apolipoproteins have a role in processing of amyloid precursor protein (APP), Aβ metabolism and neurodegenerative processes [18], [22], [41] (figure 1). The relationship between cognition and individual apolipoproteins is outlined in the following sections as it relates to the findings of this study.

### ApoA1, ApoB and Cognitive Decline

In this study, ApoA1 concentration was lower in MCI, and a low level of ApoA1 was the strongest risk factor of cognitive decline in comparison with other apolipoproteins. These observations are consistent with published studies, which have shown a marked decrease of ApoA1 levels in AD plasma and serum [21],[55] and demonstrated that decreased serum ApoA1 concentrations were highly correlated with the severity of AD [56]. Further, ApoA1 in CSF is significantly decreased in autopsy-confirmed AD patients [57]. Interestingly, a triple transgenic mouse model over-expressing mutant forms of APP, presenilin 1 and ApoA1 showed that over-expression of ApoA1 prevented the development of age-related learning and memory deficits despite continued Aβ deposition [18].

ApoA1 and ApoA2 are the principal apolipoproteins in HDL, and are responsible for the reverse transport of cholesterol, a process that removes excess cholesterol from peripheral tissues to the liver for excretion [58]. ApoA1 is also believed to be a more reliable parameter for measuring HDL than cholesterol content since it is not subject to variation [15]. ApoB represents most of the protein content in LDL and is also present in intermediate-density lipoproteins (IDL) and VLDL, and is involved in the transport of cholesterol to peripheral tissues. The ApoB/ApoA1 ratio reflects the balance between two opposite processes, and is a valuable indicator of CVD risk factors [15]. While, the roles of ApoA1 and ApoB in the periphery have been studied extensively, their functions in the central nervous system and memory function are not fully understood.

ApoB has been found to be increased in AD plasma [23] and serum [59], and our cross-sectional results are consistent with these studies. ApoB in AD serum is correlated with Aβ levels in brain [59]. ApoB is not normally found in CSF [17]. However, by using 3-dimensional immunomicroscopy, plasma ApoB was found to be co-localised with cerebral Aβ in plaques in a transgenic mouse AD model and plaque abundance was positively correlated with ApoB [17], [60]. A compromised blood brain barrier (BBB), which occurs as part of AD pathology, may allow peripheral ApoB into the brain which may then contribute to further pathology [61]. Over-expression of ApoB in transgenic mice led to significant changes in the cerebral protein profile and triggered apoptosis and neurodegeneration in the brain [22].

### ApoE and Cognitive Decline

The results in the literature on ApoE levels in AD are inconsistent. Darreh-Shori et al reported an *APOE* ε4 gene-dose dependent increase in the expression of ApoE in CSF and plasma of AD patients, with the ε4 homozygotes having about two-fold higher ApoE than non-carriers [34], [35], [62]. By contrast, the Australian Imaging, Biomarkers and Lifestyle (AIBL) study showed that ApoE levels were significantly lower in patients with AD and ApoE levels were significantly lower in ε4 homozygous individuals [29]. Other studies have also reported lower ApoE levels in AD [30], [31]. Lower serum ApoE levels were found in carriers of the *APOE* ε4 allele in another study [32]. Further investigations, particularly long term longitudinal studies, are necessary to establish if ApoE levels are potential plasma biomarkers of cognitive decline, MCI or AD. Our data showed that ApoE levels were significantly higher in the MCI group, and *APOE* ε4 homozygous carriers had significantly lower ApoE level than *APOE* ε4 non-carriers and *APOE* ε4 heterozygote carriers. However, baseline ApoE levels were not associated with cognitive decline after 2 year follow-up in this study.

The apparent inconsistencies in the published literature on ApoE levels in AD or MCI may be due to multiple differences between studies, including; (1) the sample, which can be CSF, serum, plasma, urine or tissue (2) experimental design (*i.e*., cross-sectional or longitudinal), (3) subject numbers, which can vary from a handful of individuals to many 100’s if not 1000’s of subjects, (4) methodological differences in the assay procedure which may affect sensitivity or recovery. As an example the detergent concentration in the dilution buffer may cause variation of ApoE levels measured in different studies. Furthermore, as a lipophilic protein, ApoE may be adsorbed to labware during sample handling, possibly resulting in falsely low ApoE values [33]. Standardisation of sample handling protocols would certainly improve consistency of outcomes between laboratories. However some apparent discrepancies between different laboratories may in fact reflect real differences in expression of proteins between different locations within the body, or in trends over the course of disease. For example there may be disease related differences in the expression of apolipoproteins in CSF relative to plasma or serum, due to such factors as a compromised BBB, other vascular disruptions such as cerebral amyloid angiopathy, or protein sequestration processes such as plaque deposition. Similarly, protein concentrations may vary over time and may also be differentially expressed in disease vs normal aging, which is why it is important to collect longitudinal as well as cross-sectional data, and to explore changes in prodromal conditions such as MCI and well as at more advanced stages of dementia.

### ApoH and Cognitive Decline

Our results showed that MCI subjects had a lower level of ApoH in the cross-sectional comparison, and low levels of ApoH also increased the risk of cognitive decline during follow-up. ApoH, also known as beta2-glycoprotein I (*β*2GPI), is a 44 kDa protein and highly glycosylated [63]. It circulates mainly in free form although up to 35% is associated with lipoproteins and binds to negatively charged substances, including heparin, phospholipids, and dextran sulfate. It is the main autoantigen responsible for negatively charged antiphospholipid antibodies production in the anti phospholipid syndrome (APS) [64]. ApoH may prevent activation of the intrinsic blood coagulation cascade by binding to phospholipids on the surface of damaged cells. High levels of pathogenic ApoH antibody in turn could cause hypercoagulation and venous and arterial thrombosis, and is clinically relevant to APS [65]. APS is a multi-system disorder characterized by vascular thromboses, neurological complications including vascular brain disease and increased risk of developing dementia [66]. Furthermore, an interaction between high ApoH antibody levels and amyloid plaque load in the brain was observed in a transgenic mouse model (APP_695_SWE mutation mice immunized with ApoH) [19].

Our results support current knowledge on the functions of plasma ApoH. Even though a mechanistic link between plasma ApoH and cognitive decline has not been established, it may indicate that subclinical hypercoagulation caused by low ApoH might increase the risk of cognitive decline.

### ApoJ and Cognitive Decline

Our results indicate that ApoJ plasma concentration is raised early, before AD and dementia symptoms become clinically evident. We observed that ApoJ levels were negatively correlated with global cognition and associated with white matter volume atrophy during follow-up. Apo J is a disulfide linked glycoprotein composed of two 40 kDa subunits and expressed at higher levels in the brain than in many other tissues [67], [68]. It is associated with amyloid plaques *in vivo* [69], has been shown to bind Aβ *in vitro* [70] and facilitates Aβ transport across the BBB [71]. Several studies have shown increased expression of ApoJ in the brains of AD patients [72], [73]. ApoJ in white matter is increased in aged rat brain [74]. Recently, two large genome-wide association studies revealed that the *APOJ*/clusterin gene is an important susceptibility gene for AD in different populations, in addition to *APOE* [39], [40]. Two studies demonstrated that the level of ApoJ in CSF is significantly increased in AD patients [75], [76].

We did not find that plasma ApoJ levels were associated with cognitive decline during follow-up. However, it has been reported that high plasma ApoJ was associated with clinical progression of AD [20]. The major difference between that report and our results is that cognitively normal participants in MAS have only been followed up for two years and MCI and mild dementia were the major diagnoses at Wave 2. It would be worthwhile to investigate whether ApoJ has a causal role in AD or is a contributory factor to the development or progression of the disease.

We have followed up subjects for only two years, and further follow-up will be needed to develop a more complete view of the observed relationship, especially for incident MCI and AD subjects. Serial measurements may also help determine if apolipoproteins play a major casual role in MCI and AD. The findings, moreover, need to be replicated in independent populations. The examination of transgenic animal models of AD may also be informative in this regard. The final objective would be to determine if modification of plasma apolipoprotein levels can reduce the risk of cognitive disorders in the elderly.

In conclusion, this study indicates that apolipoprotein levels are dysregulated in the plasma of MCI subjects at an early stage of cognitive decline, before a clinical diagnosis of AD is possible. ApoA1, ApoH and ApoJ may be potential clinical biomarkers for cognitive impairment. Our results provide further support for a pathophysiological role for these apolipoproteins in cognitive decline in the elderly, and possibly AD. As early indicators of cognitive decline, these apolipoproteins might also become targets of treatment or preventative healthcare measures.

## Supporting Information

Figure S1 **Scatter plot of significant correlation of transformed ApoJ and global cognition domain score.**
(TIF)

Figure S2 **Scatter plot of significant correlation of transformed ApoH and global cognition domain score.**
(TIF)

Figure S3 **Scatter plot of significant correlation of transformed ApoH and attention/process speed domain score.**
(TIF)

Figure S4 **Scatter plot of significant correlation of transformed ApoJ and attention/process speed domain score.**
(TIF)

Figure S5 **Scatter plot of significant correlation of transformed ApoE and memory domain score.**
(TIF)

Figure S6 **Scatter plot of significant correlation of transformed ApoE and verbal memory domain score.**
(TIF)

Figure S7 **Scatter plot of significant correlation of transformed ApoH and executive domain score.**
(TIF)

Figure S8 **Scatter plot of significant correlation of transformed ApoC3 and grey matter Volume.**
(TIF)

Figure S9 **Scatter plot of significant correlation of transformed ApoE and grey matter volume.**
(TIF)

Figure S10 **Scatter plot of significant correlation of transformed ApoJ and grey matter volume.**
(TIF)

Figure S11 **Scatter plot of significant correlation of transformed ApoE and CSF volume.**
(TIF)

Figure S12 **Scatter plot of significant correlation of transformed ApoJ and CSF volume.**
(TIF)

Figure S13 **Scatter plot of significant correlation of transformed ApoB/ApoA1 and hippocampus volume.**
(TIF)

Table S1 **Cognitive domains and tests used for the calculation of domain scores.**
(DOCX)

Table S2 **Ranges of standard concentrations.**
(DOCX)
