# Supplementary figures and images for: Plasma Apolipoprotein Levels Are Associated with Cognitive Status and Decline in a Community Cohort of Older Individuals
Source: PLoS One. 2012 Jun 11;7(6):e34078. doi: 10.1371/journal.pone.0034078 (PMC3372509; doi:10.1371/journal.pone.0034078)

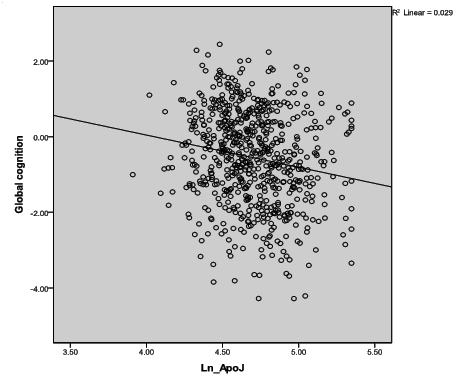

Supplement: Figure S1 — Scatter plot of significant correlation of transformed ApoJ and global cognition domain score. (TIF) [file pone.0034078.s001.tif]

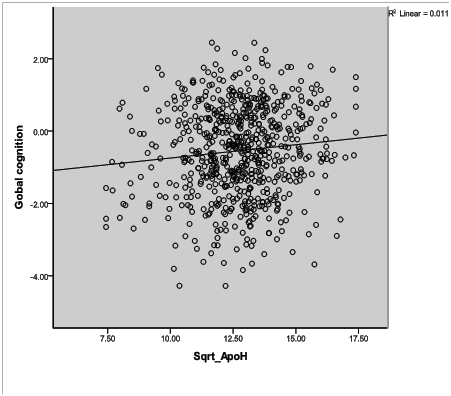

Supplement: Figure S2 — Scatter plot of significant correlation of transformed ApoH and global cognition domain score. (TIF) [file pone.0034078.s002.tif]

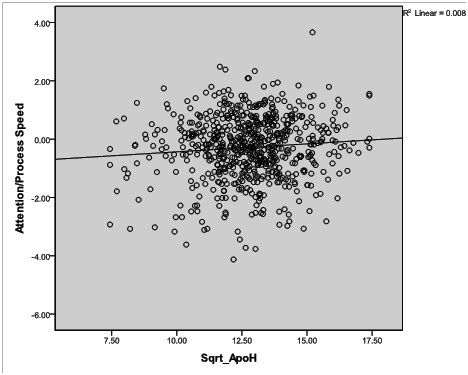

Supplement: Figure S3 — Scatter plot of significant correlation of transformed ApoH and attention/process speed domain score. (TIF) [file pone.0034078.s003.tif]

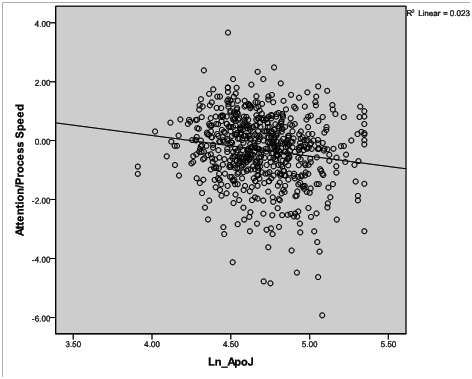

Supplement: Figure S4 — Scatter plot of significant correlation of transformed ApoJ and attention/process speed domain score. (TIF) [file pone.0034078.s004.tif]

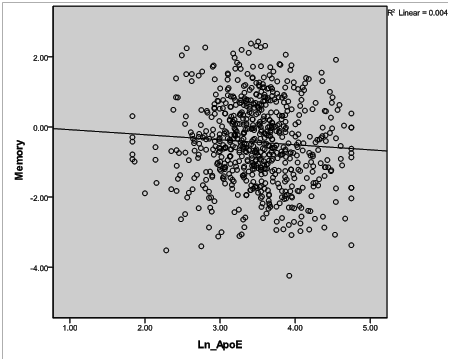

Supplement: Figure S5 — Scatter plot of significant correlation of transformed ApoE and memory domain score. (TIF) [file pone.0034078.s005.tif]

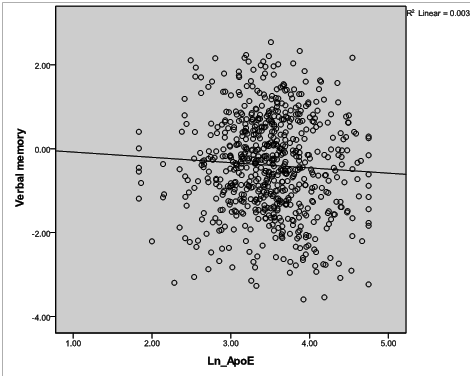

Supplement: Figure S6 — Scatter plot of significant correlation of transformed ApoE and verbal memory domain score. (TIF) [file pone.0034078.s006.tif]

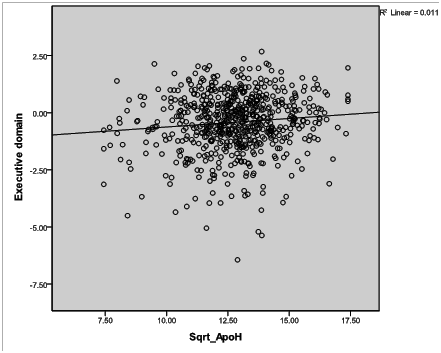

Supplement: Figure S7 — Scatter plot of significant correlation of transformed ApoH and executive domain score. (TIF) [file pone.0034078.s007.tif]

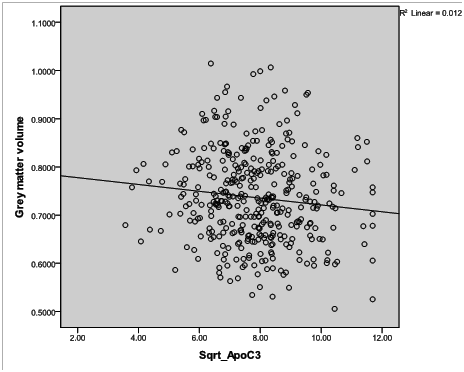

Supplement: Figure S8 — Scatter plot of significant correlation of transformed ApoC3 and grey matter Volume. (TIF) [file pone.0034078.s008.tif]

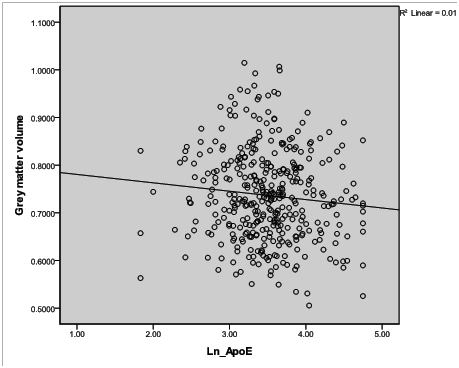

Supplement: Figure S9 — Scatter plot of significant correlation of transformed ApoE and grey matter volume. (TIF) [file pone.0034078.s009.tif]

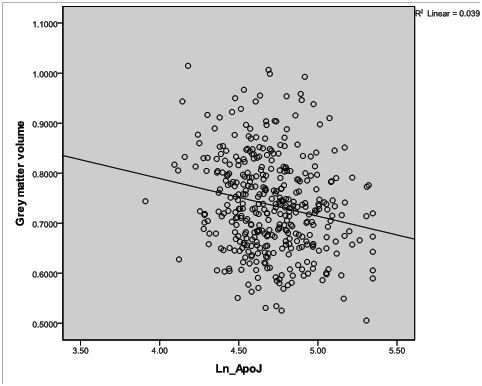

Supplement: Figure S10 — Scatter plot of significant correlation of transformed ApoJ and grey matter volume. (TIF) [file pone.0034078.s010.tif]

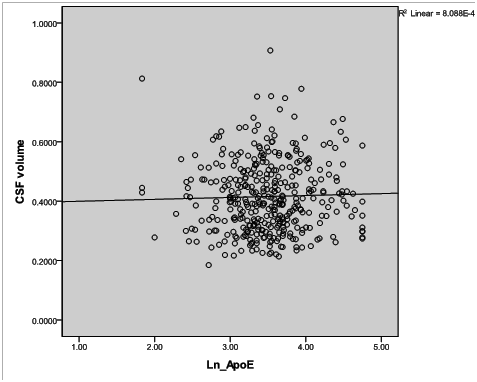

Supplement: Figure S11 — Scatter plot of significant correlation of transformed ApoE and CSF volume. (TIF) [file pone.0034078.s011.tif]

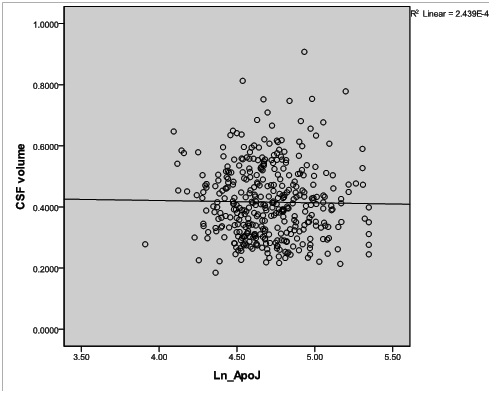

Supplement: Figure S12 — Scatter plot of significant correlation of transformed ApoJ and CSF volume. (TIF) [file pone.0034078.s012.tif]

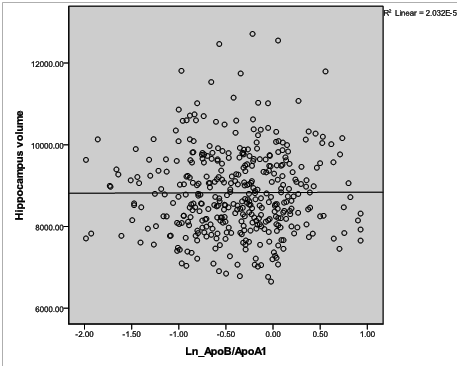

Supplement: Figure S13 — Scatter plot of significant correlation of transformed ApoB/ApoA1 and hippocampus volume. (TIF) [file pone.0034078.s013.tif]
