# Supplementary material for: Plasma Apolipoprotein Levels Are Associated with Cognitive Status and Decline in a Community Cohort of Older Individuals
Source: PLoS One. 2012 Jun 11;7(6):e34078. doi: 10.1371/journal.pone.0034078 (PMC3372509; doi:10.1371/journal.pone.0034078)
Supplement: Table S2 — Ranges of standard concentrations. (DOCX) [file pone.0034078.s015.docx]

**Table S2** Ranges of standard concentrations

|  | | **ApoA1** | **ApoA2** | **ApoB** | **ApoC3** | **ApoE** | **ApoH** | **ApoJ** |
| --- | --- | --- | --- | --- | --- | --- | --- | --- |
| Standard concentration  ng/ml | 2.0-4260 | 0.46-1000 | 2.2-4780 | 0.33-727 | 0.24-535 | 0.28-619 | 0.091-200 |  |
